# 2D Nanosheets—A New Class of Therapeutic Formulations against Cancer

**DOI:** 10.3390/pharmaceutics13111803

**Published:** 2021-10-28

**Authors:** Ravichandran Manisekaran, René García-Contreras, Aruna-Devi Rasu Chettiar, Paloma Serrano-Díaz, Christian Andrea Lopez-Ayuso, Ma Concepción Arenas-Arrocena, Genoveva Hernández-Padrón, Luz M. López-Marín, Laura Susana Acosta-Torres

**Affiliations:** 1 Laboratorio de Investigación Interdisciplinaria, Área de Nanoestructuras y Biomateriales, Escuela Nacional de Estudios Superiores Unidad León, Universidad Nacional Autónoma de México, Boulevard UNAM No. 2011, Predio El Saucillo y El Potrero, Guanajuato 37689, Mexico; rgarciac@enes.unam.mx (R.G.-C.); pserranod@enes.unam.mx (P.S.-D.); alopeza@enes.unam.mx (C.A.L.-A.); carenas@enes.unam.mx (M.C.A.-A.); 2 Facultad de Química, Materiales-Energía, Universidad Autónoma de Querétaro, Santiago de Querétaro 76010, Mexico; aruna.rasu@uaq.mx; 3 Centro de Física Aplicada y Tecnología Avanzada, Universidad Nacional Autónoma de México, Campus Juriquilla, Juriquilla 76230, Mexico; genoveva@fata.unam.mx (G.H.-P.); lmlm@unam.mx (L.M.L.-M.)

**Keywords:** 2D material, nanosheets, MXene, black phosphorus, transition metal dichalcogenides, LDHs, drug delivery, therapeutics

## Abstract

Researchers in cancer nanomedicine are exploring a revolutionary multifaceted carrier for treatment and diagnosis, resulting in the proposal of various drug cargos or “magic bullets” in this past decade. Even though different nano-based complexes are registered for clinical trials, very few products enter the final stages each year because of various issues. This prevents the formulations from entering the market and being accessible to patients. In the search for novel materials, the exploitation of 2D nanosheets, including but not limited to the highly acclaimed graphene, has created extensive interest for biomedical applications. A unique set of properties often characterize 2D materials, including semiconductivity, high surface area, and their chemical nature, which allow simple decoration and functionalization procedures, structures with high stability and targeting properties, vectors for controlled and sustained release of drugs, and materials for thermal-based therapies. This review discusses the challenges and opportunities of recently discovered 2D nanosheets for cancer therapeutics, with special attention paid to the most promising design technologies and their potential for clinical translation in the future.

## 1. Introduction

GLOBOCAN 2020, a recent report on worldwide cancer statistics analyzed from 185 countries, reveals cancer as one of the deadliest diseases worldwide, responsible for over 19.3 million (M) new incidences and causing nearly 10 M deaths in 2020. As per World Health Organization (WHO) estimation, cancer is recognized as a leading death-causing factor for those under the age of 70 years in many countries (first/second—112 ad third/fourth—23 of 183 countries). More shocking is the expected new cases projected for the year 2040, about 28.4 M, which corresponds to a 47% increase over 2020 [1,2]. Cancer is a hallmark of many other diseases, which causes several health issues among patients with various medical complications [3]. However, multiple organizations worldwide with enormous financial investments in research and development (R&D) are continuously attempting to find treatments and early diagnosis strategies for cancer with state-of-the-art technology. Continuous investments have allowed the development of therapeutic platforms against cancer; for example, one of the best-known initiatives, Cancer Moonshot, was developed in the United States in 2016 to prevent, diagnose, and treat cancer, and care for those with cancer. A recent report from this program concluded that it is merely time and investment that will result in a breakthrough cancer medicine. In the meantime, both factors will lead to researchers learning various comprehensive aspects and mechanisms regarding cancer [4].

Over the course of human history, disease treatment strategies have continuously evolved in the search for a cure or to prevent the spread of cancer, which is usually achieved by increasing the selectivity and efficacy of the drugs. The field of cancer treatment has undergone various methodologies, progress, and developments. The most common treatment is chemotherapy, where drugs are administered as a first-line treatment, but it has various drawbacks that affect the patients and lead to adverse effects. The drugs usually exhibit high toxicity, limited bioavailability, and less than ideal pharmacokinetics [5].

**Figure 1:**
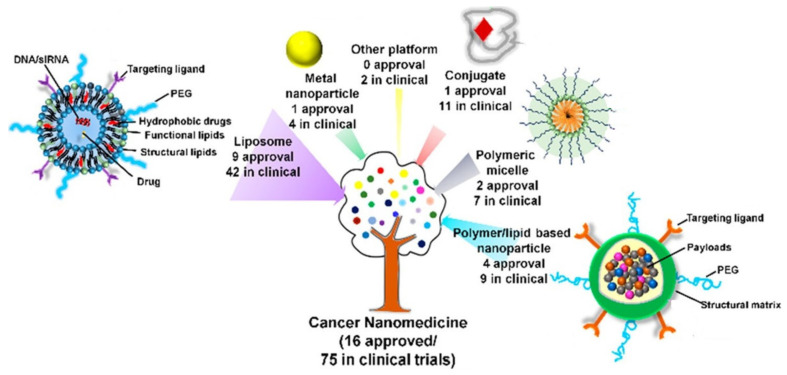
Schematic illustration of various types of cancer nanomedicine formulations—an outline of both approved and in clinical progress (adapted with permission from Reference [7], Accounts of Chemical Research, published by American Chemical Society, 2019).

The major drawback of conventional drug delivery systems (DDSs) in cancer therapy is the lack of selectivity and reduced effectiveness, which can be efficiently tackled by nanomedicine using various metallic, polymeric, and biomimetic structures. These developed structures led to the Food and Drug Administration (FDA) approval of 15 multiple nanoparticles for cancer; 75 and 190 are still under investigation and in clinical trials, respectively for DDSs [5,6,7], as shown in Figure 1.

However, the cancer therapeutics sector still requires further research; novel elements are being explored, and their combinations are being studied, because the physicochemical properties of the system play a vital role in the therapeutic results. The criteria for evaluating the therapeutic drug are nanosystem morphology, size, chemical composition, surface area, targeting ligand, encapsulating polymer, and finally, release kinetics. Apart from this, the significant challenges faced during the clinical translation of these potential drugs are controlled and sustained release, scalability, and nanostructure reproducibility. So, nanobiotechnology will be the most relevant field in the search for and production of fascinating and promising products. So far, this technology has produced different nano-based materials, some of which have entered various phases of clinical trials, for example, carbon-based, metal, metal oxide, liposomal, and polymeric formulations [8].

Graphene (G) is a well-known two-dimensional (2D) material that is a hotspot in many research areas, proving to be a promising material. Graphene has found a unique place in biomedical applications and is employed in sensing, diagnosis, imaging, antimicrobial applications, tissue engineering, and therapies [9,10]. In the past decade, various 2D nanomaterials with extraordinary properties have been discovered that are similar to G. These materials have a 100 nm plane size and have a thickness of one or more atomic layers as a freestanding sheet. The various semiconductor platforms have provided advantages in numerous fields, such as batteries, biomedical applications, catalysis, energy storage, sensors, optoelectronics, etc. [11].

G plays an important role in the biomedical field because of biologically favorable parameters such as biodegradation and biocompatibility. These conditions are desirable in cancer therapy, so G provided an opportunity for researchers to explore a wide range of possibilities. For instance, G’s unique advantages, such as thinness, provide a high surface area for tailoring or anchoring the molecule of interest, enable use as a thermal agent to ablate the tumor tissue, and provide stability in the physiological medium, and some of the elements are readily consumed by the human body. Furthermore, based on this versatility, G is often engineered for combinatorial therapy, such as chemotherapy (CTX)/photothermal therapy (PTT), PTT/photodynamic therapy (PDT), PTT/PDT/gene, PTT/gene, CTX/PTT/gene, CTX/PTT/PDT, etc., as a highly multifunctional formulation [12]. So, 2D materials in cancer research have become a hotspot and are being extensively investigated for mono and synergistic therapies [13]. In recent years, several 2D materials have been discovered for use in cancer nanomedicine, such as transition metal dichalcogenides/dioxides, black phosphorous, graphitic carbon nitride, MXenes, layered double hydroxides [14,15], covalent–organic and metal–organic frameworks, palladium and manganese dioxide, boron nitride, pnictogen (phosphorene, arsenene, antimonene, and bismuthine), and nanosheets [13,16].

**Figure 2:**
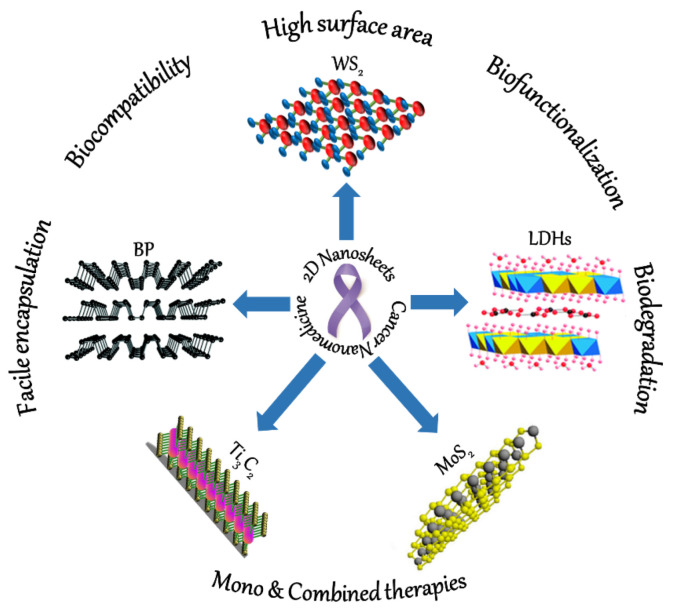
Schematic illustration of the different 2D NSs and their advantages for versatile cancer therapeutics. BP (adapted from Reference [17], Materials Horizons, published by The Royal Society of Chemistry, 2017), Ti_3_C_2_ (adapted with permission from Reference [18], Journal of Molecular Liquids, published by Elsevier, 2020), MoS_2_ (adapted with permission from Reference [19], Langmuir, published by American Chemical Society, 2017), WS_2_ (adapted from Reference [20] under the terms of the Creative Commons Attribution 4.0 International License), and LDHs (adapted from Reference [21], RSC Advances, published by The Royal Society of Chemistry, 2014).

Even though numerous 2D materials are currently being used for various biomedical applications, this review specifically provides an update on the recent research advances in the widely investigated nanosheets (Ti_3_C_2_, BP, MoS_2_, WS_2_, and LDHs) that show promise for cancer therapeutics (Figure 2). Even though there are handful of review articles on 2D NSs on cancer, they only provide a wide overview of all the therapies. So, this review is dedicated purely to the field of drug delivery, which is considered to be the first-line treatment approach for any cancer type. So, we chose the 2D materials and their design to provide reader san overview of various site-targeting and encapsulation technologies (polymeric, cell-membrane based, hydrogel, and liposome) used to a novel formulations. This review describes the different 2D-based cancer therapeutic formulations by explaining the importance of drug loading and release for mono or combined therapies. Additionally, we demonstrate the interface of drug internalization in the tumor region, discussing outcomes such as decrease in and/or inhibition of tumor growth with no recurrence.

All the described nanosheets (NSs) were designed and are modified innovatively based on their treatment of various cancer types. Therefore, we provide a brief outline of the properties and advantages of each NS to provide readers with the current strategies followed by the scientists. In most cases, NSs are used for combined therapies to release drugs in a specific region by various targeting ligands to avoid the bottlenecks experienced by most of the conventional treatments. In general, our focus is on the therapeutics, as we found that monotherapies, such as pH- and stimuli response-based methods, release a very minimal amount of drugs. So, most of the work described herein applied the principle of multimodal therapy (CTX/PTT), which releases an ample concentration of drugs that leads to the killing of tumor cells with no recurrence. Finally, we describe our perspectives on the various challenges and future outcomes of 2D-NSs-based cancer therapeutics.

## 2. 2D Nanoformulations for Drug Delivery

### 2.1. MXene Nanosheets

MXenes are ultrathin 2D layers of graphene-like morphology composed of vast amounts of metal nitrides, carbides, and carbonitrides with a formula of M_n_ + _1_X_n_, where M, n, and X represent the element of the transition metal, the number of M layers, and carbon/nitrogen. This novel nanomaterial inherits outstanding properties that can be utilized for various biomedical applications. These MXenes can be synthesized using top-down and bottom-up approaches, resulting in different nanomorphologies based on the applications. Researchers have explored some of the 2D MXene NSs, including titanium carbide (Ti_3_C_2_), niobium carbide (Nb_2_C), and tantalum carbide (Ta_4_C_3_), searching for a multifunctional behavior that can be used in cancer nanomedicine. Therefore, in this section, we chose one of the widely investigated MXenes, Ti_3_C_2_, and report its recent advances with various modifications and strategies to enable its use as a drug delivery platform for cancer [16,22,23].

#### Titanium Carbide (Ti_3_C_2_) Nanosheets

**Figure 3:**
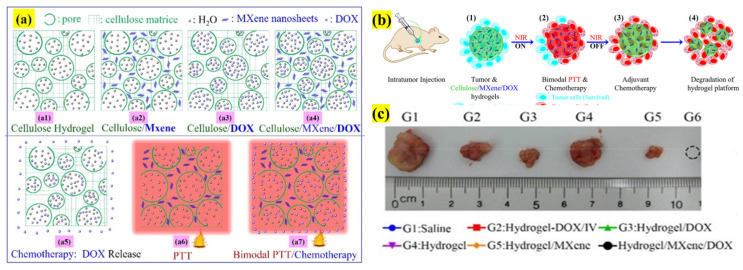
Schematic illustration of the design and 3D hydrogel morphology composed of cellulose, NSs, and DOX for bimodal CTX/PTT-based cancer treatment. (**a**) Four kinds of gels: (**a1**) cellulose hydrogel, (**a2**) NSs-integrated matrix, (**a3**) DOX-loaded matrix, and (**a4**) NSs/DOX-containing hydrogel; (**a5**) DOX-CTX; (**a6**) NSs-based PTT; and (**a7**) bimodal CTX/PTT therapies. (**b1**–**4**) Graphical representation of in vivo CTX/PTT therapy using cellulose hydrogel matrix; (**c**) tumor size representative for each group. (Adapted with permission from Reference [24], ACS Applied Materials & Interfaces, published by American Chemical Society, 2018).

Ti_3_C_2_ NSs have been widely investigated, providing various functionalities in anticancer therapy that overcome various limitations and challenges. Xing et al. proposed a three-dimensional (3D) cellulose hydrogel composite of NSs and doxorubicin (DOX) to increase the stability, biocompatibility, localized, and sustained DOX release of monotherapies or combined therapy (CTX/PTT), as presented in Figure 3. The 3D hydrogel has high water retention (98.5 wt %) and a porous structure, and efficient DOX loading was achieved based on the NSs ratio. For instance, 0.69 mg/mL was loaded for 235.2 ppm. The hydrogel exhibited a 54.7% strain and 23.8 kPa compressive force with slight cracks, indicating high suitability for injection. The DOX liberation was slow without illumination but, surprisingly, there was an intense increase in the release pattern in the surrounding medium after near-infrared (NIR) light irradiation. This might be due to the expansion and swelling of hydrogel pores under the influence of irradiation, which causes the gel to sink in the medium and enhances the release. Mice inoculated with hepatocellular carcinoma were injected with hydrogel, and combined therapy with various control groups was tested for 2 weeks. At the end of the experiment, it was found that combined CTX/PTT therapy resulted in complete eradication with no relapse of cancer. The temperature increase of NSs induces the release and degradation of DOX in the cells, thus demonstrating the potential of the hydrogel platform for cancer therapeutics [24].

Synergistic applications of Ti_3_C_2_ NSs have often been explored by developing a versatile all-in-one platform for cancer therapy aiming at tumor suppression. In one work, NSs were initially synthesized using the two-step exfoliation method, and encapsulated with phospholipid from soybean to enhance their stability and dispersibility. Then, DOX was surface-functionalized on the NSs, resulting in a size of 164.2 nm with a zeta potential of −28.9 mV. These parameters are favorable for facile blood circulation and accumulation into the tumor region, with a DOX loading capacity of 211.8%. The drug-release studies at three different pH levels (4.5, 6, and 7.4) showed that 58%, 33.9%, and 14.2% of the drug was released, respectively. In the NIR illumination, the DOX liberation at pH 4.5 increased to 81.5% in a 12 h period.

The incubation of functionalized NSs with 4T1 cancer cells treated with CTX, PTT, and combined (CTX/PTT) therapies showed 39.5%, 52.8%, and 74.6% cell inhibition rates, respectively. The animal studies showed an accumulation of 2% and 3.6% at 4 and 24 h, respectively. The PTT increased the temperature to 68.5 °C and enhanced DOX release, which could ablate a tumor. Combined therapy resulted in complete eradication. The size of NSs aids in easy excretion after 48 h and 18.7% and 10.35% of the drug were found in urine and feces, respectively. Thus, the designed nanosystem proved to be highly biocompatible and stable, with drug loading efficiency and a desirable therapeutic outcome. Therefore, this system provides a new path in 2D nanomaterial nanomedicine [25].

**Figure 4:**
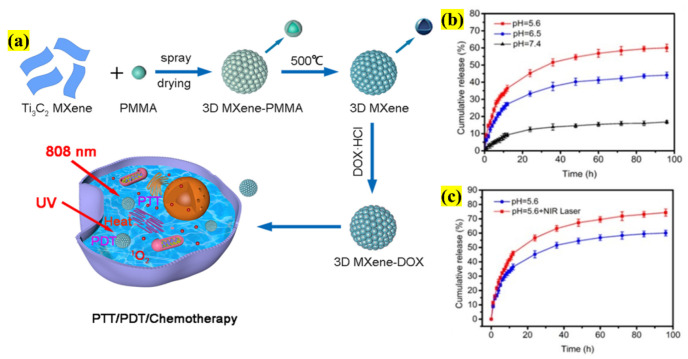
Schematic illustration representing (**a**) the fabrication of a 3D NS microsphere using PMMA and loading with DOX for synergistic CTX/PTT/PDT treatment of a tumor; (**b**) cumulative drug liberation at various pH values; (**c**) DOX release after NIR irradiation (adapted with permission from Reference [26] (2021) © IOP Publishing. All rights reserved).

In a recent study on NSs, the authors developed a 3D honeycomb structure to overcome adverse effects such as size distribution and agglomeration. So, the authors constructed a novel strategy by coupling conventional materials with modern fabrication technology. Initially, the NS surface was oxidized by spray-drying poly(methyl methacrylate) (PMMA), leading to the formation of a hard template. After creating a 3D microsphere, the shell was removed by calcination, resulting in a size of 3 µm (Figure 4). The DOX loading reached a maximum of 87.3% because of the microsphere structure (pore size of 2–8 nm), and was finally employed in combinatorial therapies. The stimuli-based drug release response was studied; for example, at pH 5.6 and 7.4 for a period of 96 h, 60% and 17% were released, respectively. With NIR irradiation, the DOX release was enhanced, confirming the microspheres release behavior under stimulation. Finally, the combinatorial therapy (CTX/PTT/PDT) was applied in the presence of HeLa cells, where 500 μg/mL 3D NSs-DOX showed a cell-killing response of 13.2%, which is considerably higher than that of the monotherapies, which was 27.4% under UV light and 18.5% under the influence of a NIR laser, thus confirming the potential of this novel architecture for synergistic therapy [26]. Other similar studies were conducted using NSs for trimodal (CTX/PTT/PDT) therapies, as denoted in Table 1.

### 2.2. Black Phosphorous (BP) Nanosheets

Phosphorus (P) is an essential element for the human body, which constitutes around 1–1.4% of body weight and plays a vital role in several biological functions, such as in the synthesis of adenosine triphosphate (ATP), deoxyribonucleic Acid (DNA), cell membrane synthesis, protein phosphorylation, and B vitamins, which contribute to the function of kidneys, nerve signaling, muscle contractions, etc. [27]. P exists in three different allotropic forms, white, red, and BP, which have distinctive applications. BP, also known as phosphorene (monolayer form), received considerable attention from scientists in 2014 after its first production by exfoliation because of its unique electronic, optical, mechanical, and thermal properties, and structure. It has a honeycomb structure composed of many twists and turns of P atoms bound by an inter- and intralayer of van der Waals forces and P–P bonding, respectively [28]. So, in the last few years, it has been applied in sensing, therapeutics, photocatalysts, etc. Considering its biomedical applications compared to other 2D nanomaterials, BP has been widely accepted due to its high biodegradability, compatibility, photostability, and low cytotoxicity, making it outstanding amongst the rest [29].

BP, in the field of cancer theranostics, is widely utilized in PTT due to its larger extinction coefficients and high NIR photothermal conversion efficiency; in PDT due to the capability to be used as a photosensitizer as it efficiently generates singlet oxygen; and in CTX because of its fold and wrinkle structure and the large surface area, which provides a large platform for biofunctionalization of large quantities of antineoplastic drug moieties or imaging agents. This section reviews BP NSs as effective drug delivery carriers in detail by considering some recent advances in DDSs. Usually, single- or multi-layered BP NSs are produced by various exfoliation methods, such as mechanical and liquid-phase methods, including organic solvents, water, and ionic-liquids-assisted methods. The functionalization can be achieved through three different strategies: covalent or noncovalent bonding and electrostatic interactions, which are based on various parameters [30].

Research on BP NSs in DDSs is still in its infancy, and the bottleneck lies in the synthesis of sheets, which is quite complicated. For instance, chemical exfoliation methods involving harsh chemicals, reaction conditions, time-consuming post-treatments such as purification and modification are necessary. So, Poudel et al. developed a continuous batch-by-batch process using catalytic conversion to synthesize BP NSs without the aforementioned steps. In this investigation, the all-in-one precursor of red P, iodine, and gold–tin were used to synthesize NSs, which were encapsulated with layer-by-layer (LbL) assembly of DOX, provided a positive surface charge by poly-L-lysine (PLL), and finally a cluster of differentiation (CD)-44 overexpressing cells-targeting agent, hyaluronic acid (HA). The functionalized NSs as DDSs against breast cancer were assessed in both in vitro (MCF-7 and MDA-MB-231) and in vivo (MDA-MB-231 xenografted mice) studies. The in vitro DOX release after 24 h at two different pH values (5.5 and 7.4) showed a cumulative release of ~77% and ~59%, respectively, based on the pH-responsive and site-specific release. In a xenograft model, the DOX release with NIR irradiation produced a substantial decrease in tumor size with increased accumulation, resulting in drastic changes in the region, causing cellular necrosis, fragmentation, and apoptotic condensation compared with the control BP. Thus, this BP complex showed its potential and the new horizons of BP-based formulations in cancer treatment [31].

**Figure 5:**
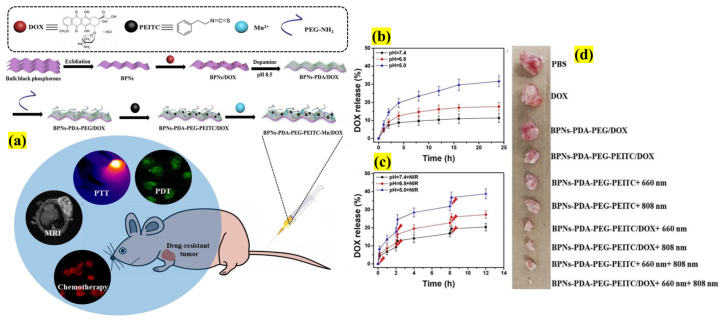
Schematic illustration showing (**a**) the multi-step synthesis of NSs with polymer (PDA-PEG) modification along with Mn and DOX loading to treat ADR cancer treatment, (**b**) pH-based DOX release profiles from the designed complex, (**c**) drug release under the external stimuli of NIR irradiation, and (**d**) tumor photographs after extraction from various treated groups (adapted with permission from Reference [33], Chemical Engineering Journal, published by Elsevier, 2019).

Even though CTX is considered the first-line treatment for many metastatic cancers, it use still experiences significant challenges because the p53 gene mutation causes resistance, leading to multiple drug resistance (MDR). Thus, currently, a novel strategy should be developed to overcome the MDR effect and to decrease/inhibit the levels of mutant p53. p53 protein levels can be selectively depleted by the inherent property of phenethyl isothiocyanate (PEITC) [32] and its anticancer effect can be enhanced by conjugating with 2D BP NSs (Figure 5). In this study, the authors designed multifunctional NSs comprising BP, DOX, polydopamine (PDA), and aminated polyethylene glycol (Am-PEG), functionalized with the PEITC polymerization method. Interestingly, because of BP NSs’ high surface area, the DOX loading was found to be ~233% and its efficiency was demonstrated in both in vitro and in vivo testing using adriamycin (ADR)-resistant breast cancer cell lines. In both the assays, the cumulative drug release amounts were augmented by NIR irradiation because of burst release, proving it to be both a pH- and stimuli-responsive carrier. To determine the function of PEITC, a Western blot assay was carried out, which showed that p53 expression was inhibited without affecting the viability of cells. Furthermore, the p53 levels considerably decreased compared with the control samples without PEITC. This demonstrated that the decorated BP NSs may be more suitable for treating various MDR cancers [33].

The synthesis and stability of BP NSs are complicated, as BP NSs mostly need post-treatments to use them effectively in biomedical applications. For instance, when NSs encounter air or water, compositional degradation occurs, thus affecting the materials’ optical properties. To address this problem, Liu et al. developed a novel platform without a functionalization process by employing platinum-based drugs to stabilize the BP NSs and used them directly for drug-release studies. Drugs such as cisplatin (CIS) and oxaliplatin (DACH) in various ratios were used to stabilize NSs and PEG encapsulation. The stability of the complex was evaluated, and the drug-stabilized BP NSs were found to be highly stable (90% after 168 h) compared with pristine NSs, which degraded quickly and continuously (53% at 24 h and 42% at 168 h). Therefore, the stabilized complex was further investigated for drug release applications using HeLa and A549 cell lines. Combined with PTT, the drug release was higher after 48 h (16% at pH 7.4 and 35.4% at pH 5) in in vitro studies. Furthermore, as a synergistic PTT/CTX therapy, the stable complex effectively suppressed all the cancer cells in the xenograft model, thus confirming it to be a promising concept for the high loading and stabilizing of NSs for cancer therapy [34].

Liposomal formulations have attracted keen interest amongst researchers for drug encapsulation and delivery. Specifically in the field of cancer, studies of liposomes significantly increased after the FDA approval of doxil. The major advantage of liposomes is that they can be tailored and functionalized based on administration and disease type, being one of the effective formulations approved in recent years [35,36]. However, few studies have been conducted using the liposomal formulation developed using BP NSs, which is discussed here.

**Figure 6:**
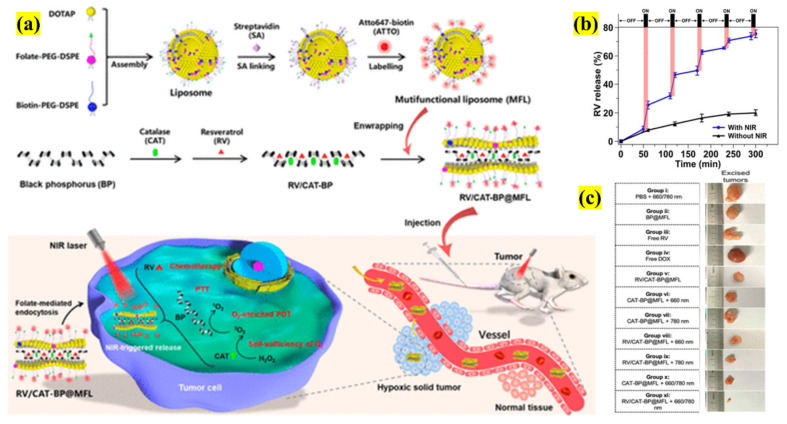
Schematic illustration depicting the (**a**) synthesis of RV/CAT functionalized NSs-liposome as a PT-responsive DDS and a PDT agent for cancer therapy, (**b**) RV release after 780 nm NIR laser trigger, (**c**) photographs of excised tumor tissues from representative mice of different groups after treatment (adapted with permission from Reference [37], ACS Applied Nano Materials, published by American Chemical Society, 2020).

Different methods than those mentioned above to produce liposomal formulations have been reported, but they lack the ability to be translated to the clinical translation in most cases. So, a facile and innovative liposomal formulation was developed by Hai et al., in which a multifunctional system was fabricated using PTT transducing agent BP NSs, anticancer drug resveratrol (RV), and PDT agent catalase (CAT), which is wrapped targeting ligands (folate-FA) and PEG-ylated liposome (Figure 6). In this study, the drug release was based on NIR irradiation using PTT to enhance the liberation and sustain the cancer cells.

A NIR laser triggered RV release from 8.4–25.4% over a duration of 1 h, which was interpreted as burst release, and finally, at the end of 5 h, more than 75% of RV was released from the system. The advantage of combined PTT/CTX therapy showed greater therapeutic effects than monotherapies. Due to FA targeting, the major BP NSs complex accumulation occurred in the tumor site 60 min post-injection. Based on these results, an in vivo study with different controls was used to examine their impacts. PTT/CTX therapy showed an increased therapeutic effect compared with the free drugs and other controls. Remarkably, the trimodal PTT/CTX/PDT therapy demonstrated a remarkable anticancer impact. Thus, this liposomal BP NSs formulation, with high stability, loading efficiency, biocompatibility, and negligible toxicity, was found to be a promising complex for clinical translation [37].

Based on a 2020 cancer statistics report, prostate cancer (PC) accounts for the second most common cancer in men [38]. Even though late-stage PC can be treatable with conventional therapies, these therapies fail in more than 50% of the patients due to various challenges. So, there is a necessity to find an efficient formulation to effectively treat PC. BP NSs utilization in cancer theranostics paved the way for Li et al. to design a novel platform for PC using zinc ions (Zn^2+^) and targeting aptamer for combinatorial therapy. The Zn^2+^ stabilizes the BP NSs and helps inhibit the activity of enzyme mitochondrial aconitase to main healthy functions of the prostate. The aptamer chosen for this study was AS1411, which can bind with high affinity to nucleolin on PC cells. The authors synthesized a complex of BP-AS1411 with Am-PEG and conjugated with Zn^2+^ with the loading of DOX. The drug release at pH 5 was 43%, and at pH 7.4, it was almost half (22%) in about 24 h. However, PTT/CTX treatment liberated more than 56% of DOX (pH 5, 24 h), proving the NIR-light-induced release hypothesis. In vivo studies using combination therapy determine the effect of Zn-stabilized BP NSs proved them to be highly efficient in suppressing cancer cell growth; most importantly, some of the animals were completely cured of cancer after treatment for 16 days. This proves the enhanced anticancer effect of NSs–Zn-based formulations for possible PC treatment and opens a new line of research [39].

Polymer-based DDSs are constantly being developed for sustained and controlled drug release to enhance the characteristic behavior and improve degradation rate, time, stealth, etc., to enhance therapeutic efficacy. Thus, most research has currently focused on smart hydrogels, which release the drug under external stimuli [40]. Therefore, NIR-light-triggered NSs of agar-based hydrogel were fabricated to release drugs and induce apoptosis in the cancer cells. First, the DOX release was measured using UV–Vis spectroscopy; as the hydrogel is NIR sensitive, the drug release profile depends on the incident light time. For instance, the drugs released were directly proportional to the irradiation time of the gel, and the visible or ambient light did not affect liberation. Then, in animal studies, mice bearing tumors were exposed to 808 nm NIR laser irradiation (1.0 W/cm^2^) for 5 min. They were then monitored for various times (1, 12, and 24 h); the effects were found to be site-specific and release was sustained after 12 h without affecting other cells. Finally, the tumor size was compared until 14 days with other controls: BP NSs hydrogel treated mice demonstrated an extremely small tumor size without any side effects or damage to the major organs, thus proving the hydrogel to be very sensitive for drug delivery applications with no toxicity, extending its application possibilities [41]. Some other studies that have designed NSs for mono and combined therapies are listed in Table 1.

### 2.3. Transition-Metal Dichalcogenides (TMDCs)

Semiconductor TMDCs sparked interest in various fields in the last decade because of their distinctive optical, electrical, and mechanical properties. TMDCs represent the analogues of 2D graphene and have created a massive niche in almost all fields. TMDCs are usually denoted by the formula MX_2_, where M represents the transition metal, such as Mo or W, and X represents the chalcogen, such as S, Se, or Te [42]. This gave rise to the possible combination of molybdenum disulfide (MoS_2_), tungsten disulfide (WS_2_), molybdenum diselenide (MoSe_2_), and tungsten diselenide (WSe_2_) structures, which have been widely investigated in fields from electronics to biomedical applications. In this section, we chose two of the most studied NSs (MoS_2_ and WS_2_) in the field of multifunctional cancer theranostics, which have enormous prospects for surface functionalization with different targets, imaging agents, drugs, or fluorescent markers enclosed in a polymeric/biomimetic structure. Its function have been tested both in vitro and in vivo, proving the NS platform’s capabilities.

#### 2.3.1. Molybdenum Disulfide (MoS_2_) Nanosheets

Mo is a biologically important constituent for the healthy human body as it plays a vital role as an enzyme cofactor for the oxidase of sulfite, xanthine, aldehyde, and mitochondrial amidoxime reducing components [43].

Among the TMDCs, MoS_2_ is a widely studied NS and has become a hot research topic, especially in cancer theranostics. MoS_2_ NSs have gained the interest of many researchers because of their unique optical and electronic properties, graphene-like planar structure, large surface area, facile bio-functionalized surface, high absorption rate, and high biocompatibility compared to other NSs. However, one of the significant shortcomings is that pristine MoS_2_ NSs have less stability and dispersibility in water; thus, in most cases, researchers have aimed to find various functionalization approaches to effectively use it for administration in the human body. Therefore, over the past few years, the use of MoS_2_ NSs in cancer treatment and therapy has been overwhelming. So, in this dedicated section, we chose to review some of the recent advances and their importance in cancer drug delivery [44,45,46].

The pioneering work on MoS_2_ NSs was carried out by Chou et al., which demonstrated the efficient capacity of NSs and their potential to absorb NIR, which paved the way for various current research lines [47]. However, even though various nano-based DDSs for cancer have been researched, only few nanocarriers have entered clinical trials due to the multiple drawbacks of the in vivo model, where the drug accumulates less in the targeted tumor region due to enhanced permeation and retention (EPR), and excess ends up in the other vital organs. The MoS_2_ NSs also face a similar scenario; thus, novel and facile techniques have been developed. Wang et al. designed an implant prepared in oleosol using an FDA-approved poly(lactic-co-glycolic acid) (PLGA) that comprises NSs and DOX. The significant advantage of this oleosol is its in situ administration enabled by high syringeability in an invasive manner. The implant was loaded with 95% of DOX and evaluated for pH- and NIR-responsive release. The in vitro pH release at 5.4 and 7.4 was roughly 18.9% and 9.4%, respectively, after 96 h due to PLGA encapsulation, which prevented drug liberation in the surrounding medium. Interestingly, when the implant was subjected to NIR irradiation (5 min), the drug release was enhanced in the acidic medium (8.7% to 31.8%). This is attributed to heat generation from the NIR laser, which triggered the drug release. Tumor-bearing mice were implanted and treated using combined therapy, which resulted in complete tumor disappearance with no recurrence. The significant advantage of this implant was that DOX release was site-specific, with <1% being found distributed in other organs. Therefore, the implant demonstrated its possibility for clinical translation because of its advantages along with localized therapy [48].

Clinically, surgery and chemotherapy seem to be the most common treatments offered for cancer. However, despite significant advances in this area, increased incidence and mortality are progressively resulting in decreased success rates. This is due to multidrug resistance (MDR) of cancerous cells, which is a clinician’s nightmare in a short treatment time, causing more than 90% of patient deaths. So, currently, nanomedicine is tackling this issues specifically to improve a patient’s quality and duration of life [49,50].

**Figure 7:**
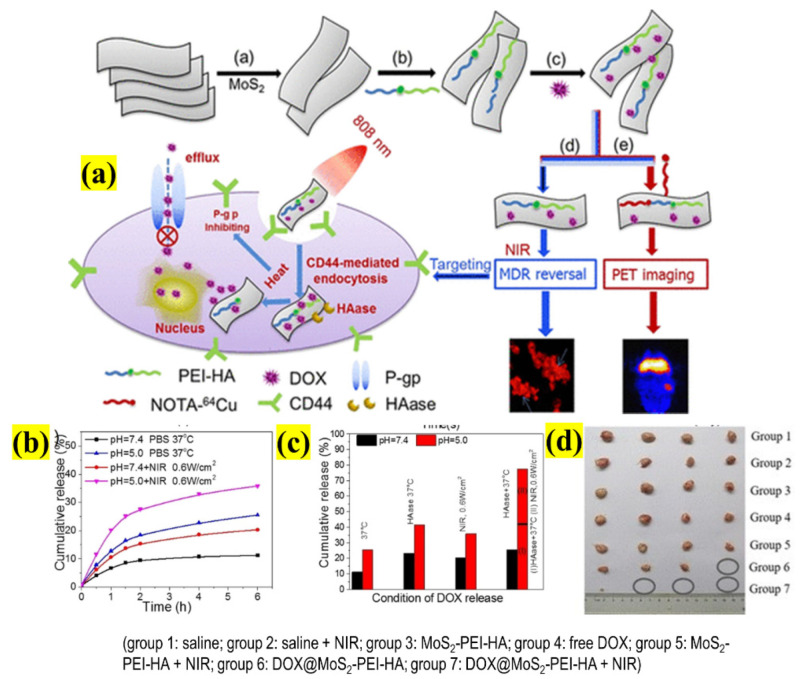
Schematic illustration representing (**a**) multifunctional NSs with surface functionalization of PEI and HA, and DOX to treat ADR by selective targeting and stimuli-based therapy; (**b**) DOX release curve from functionalized NSs with or without laser irradiation; (**c**) statistical data of percent of cumulative DOX release for various values of pH value, enzyme, and NIR laser; (**d**) excised tissue photographs of multiple treatment groups (adapted with permission from Reference [52], ACS Applied Materials & Interfaces, published by American Chemical Society, 2018).

Therefore, there is an urgent need to develop a multifunctional nanosystem to overcome the MDR caused by the overexpression of P-glycoprotein (Pgp) [51]. A facile and versatile nanosystem was developed using polyethyleneimine (PEI)-modified MoS_2_ NSs surface-functionalized with HA to effectively deliver DOX and reverse MDR in drug-resistant breast cancer (MCF-7-ADR), as shown in Figure 7. At pH 8, the pH-dependent DOX loading was >33%. This nanosystem is a multiple-stimuli-responsive carrier, where DOX is released on three bases: pH, HAase, and NIR irradiation. At 6 h, in the presence of HAase, the DOX was 41.65% and 23.2% at pH 5 and 7.4, respectively. When combined with PTT (without HAase), the drug release seemed to be pH-dependent, and at pH 5, it increased from ∼25.5%to 35.8%; finally, with HAase, it reached 77.4%. When the complex was incubated with cells, the viability extraordinarily decreased to 2.9% in the earlier condition. The Western blot experiment confirmed the reduced expression of Pgp in the cells in the presence of NIR. After various confirmations of the potential applications of the complex, the authors tested the complex in MCF-7-ADR-inoculated mice. The experiment results were obtained 25 days post-treatment; the tumors in the control groups were not affected, whereas the complex without and with NIR irradiation produced 76% and 96% tumor size reductions, respectively, with almost complete elimination and no regrowth of cancer. Thus, this design could effectively be employed in MDR-based cancer therapeutics [52].

**Figure 8:**
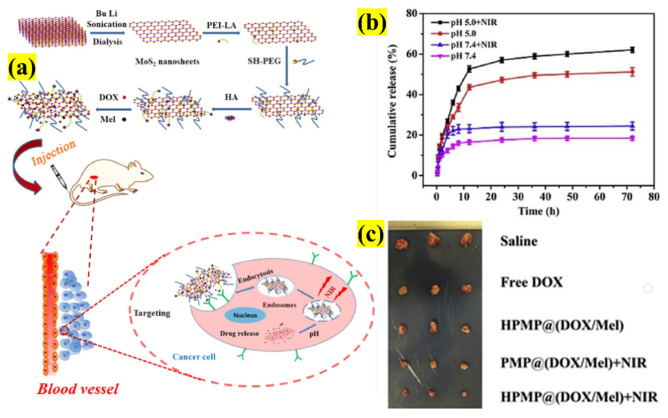
Schematic illustration showing (**a**) the DOX- and Mel-loaded NS platform as a DDS and synergistic CTX/PTT treatment of tumors, (**b**) release profiles of DOX with or without 808 nm laser at various times and pH values, and (**c**) photographs of the dissected tumors at the end of the treatment of the mice (adapted with permission from Reference [53], Colloids and Surfaces B: Biointerfaces, published by Elsevier, 2020).

Designing a multifunctional system for cancer theranostics is an ongoing quest that involves numerous components that are engineered the enable the more efficient use of the drug, thereby addressing monotherapy’s drawbacks. Yang et al. proposed a novel nanocarrier composed of PEI-modified NSs decorated with HA, encapsulated in polyethylene glycol (PEG), to target overexpressed CD44 and MCF-7 cells. In addition, they used DOX, an anticancer drug, and melanin (Mel), a PTT agent, to further enhance the drug release toward the cells (Figure 8). As a result, a composite 104 nm in size was synthesized with an increased DOX loading (944.3 mg/g), biocompatibility, and finally, an outstanding PTT efficiency of 55%. Initially, in vitro drug release was studied at pH 5 and 7.4, which was 12% and 5% after 1 h, respectively; with and without NIR irradiation at pH 5 at 6 h was found to be increased from 24.8% to 31.6% under the influence of an 808 nm laser (1.5 W cm^−2^), thus proving that the designed composite can be used for a dual stimuli (pH/NIR)-based carrier.

The cellular uptake was evaluated, which confirmed that the incorporation of Mel increased the uptake with the incident NIR laser utilizing receptor-mediated endocytosis. Based on this promising result, an in vivo study was conducted, in which the cell growth was inhibited and the MCF-7 cells were efficiently killed with negligible side effects. In conclusion, nanosystem design plays a vital role in effective targeting and therapy, demonstrating the CTX/PTT complex’s synergistic effect [53]. Similarly, we found that other NSs encapsulated with polymeric membranes and functionalized with drug molecules have been employed for dual-modal therapy (CTX/PTT), as presented in Table 1.

#### 2.3.2. Tungsten Disulfide (WS_2_) Nanosheets

Tungsten-based TMDCs have received tremendous interest after the first-reported synthesis on different nanostructures in 1992 [54]. Thus, in this section, recent advances and the biological applications of WS_2_ in the field of cancer therapeutics are discussed in detail. WS_2_ is a graphene-like material with a large surface area, providing an enormous platform for biofunctionalization of the surface with multifunctionalities and increased drug loading capability, NIR absorption, and biocompatibility, facilitating its use in diverse biomedical applications [55,56]. It can be widely used for synergistic drug release in site-specific cancer regions along with PTT. However, even though WS_2_ has been widely investigated, DDSs are still in the infant stage because of the bottlenecks such as poor solubility, requiring post-synthesis treatments, etc. So, researchers are tackling these issues using various strategies to achieve its 100% usage in DDSs. For instance, biomimetic approaches are often employed in cancer therapeutics to enhance drug concentration, tumor accumulation, stimuli-based DDSs release, etc. [57,58].

The liposomal-based DDSs formulations are an hot topic in the field of bio-nano hybrid therapeutics because of their facile conjugation techniques, biocompatibility, and controlled/sustained drug release properties [59]. Liu et al. investigated the interaction mechanism of liposomes with MoS_2_ and WS_2_ to use them in drug delivery applications. Phosphocholine (PC) lipids were employed to encapsulate TMDCs to study their adsorption capacity, intactness, stability, drug loading, cellular uptake, etc. Initially, the authors synthesized both TMDCs 50–200 nm in size, which is considered an optimal size for the EPR effect. After PC coating using the fluorescence technique, the absorption was assessed: WS_2_ (400 μg/mL) adsorbed 70%, whereas MoS_2_ (500 μg/mL) adsorbed only ~60% of the liposomes. Thus, further studies were carried out only on WS_2_, which showed higher PC adsorption than MoS_2_, and the DOX loading capacity of WS_2_ was ~30%. After DOX loading with WS_2_, the PC attachment increased to 110% compared to only WS_2_. Additionally, using various reagents, the PC attachment onto the surface of WS_2_ was found to be stable with van der Waals forces. However, this study did not perform drug delivery but instead provided a brief interaction methodology with lower cell viability. Therefore, this study provides the basis for future investigations and developments [60].

In one of the studies, WS_2_ NSs were surface-functionalized with liposomes and DOX loading to determine their effect on pH- and PTT-based drug release characteristics. The synthesized WS_2_ was about 5–6 nm thick, and after liposome formulation, the size increased to 10–15 nm with an 87% DOX loading capacity and high dispersibility. The in vitro pH release was studied for 8 and 168 h, where the cumulative release at pH 5 and 7.4 was approximately 22% and 13% and 43% and 32%, respectively. For PTT release after 4 h, pH 5 showed enhanced DOX liberation (45.7%) compared with CTX. When combining CTX and PTT, the cell survival rate was 20%, demonstrating that the combination treatment ha higher efficacy. In an in vivo study with subcutaneous injection of 4T1 cells, the enhanced internalization aided in a temperature increase of >25 °C, leading to the killing of cancer cells, thus proving the developed formulation was highly effective with increased dispersibility, stability, and cellular uptake, paving the way for a new line of research in cancer nanomedicine [61].

**Figure 9:**
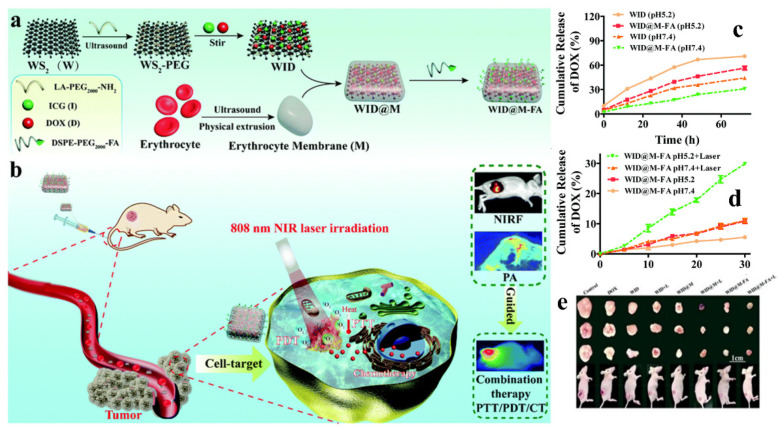
Schematic illustration depicting (**a**) the synthesis and preparation method of multifunctional RBC and LA-PEG membrane-coated FA-functionalized NSs and (**b**) synergistic drug release behavior of CTX/PTT; (**c**) DOX release curve with and without FA functionalization on NSs at pH 7.4 and 5.2; (**d**) DOX release profile with or without NIR laser irradiation; (**e**) digital photographs of tumor tissues from each group after 18 days’ treatment (adapted from Reference [63], Biomaterials Science, published by The Royal Society of Chemistry, 2020).

Recently, the usage of the cell membrane as a biomimetic system has been widely explored to overcome the EPR effect, enhance blood circulation time and site-specific targeting, and achieve immune escape [62]. Long et al. designed a versatile biomimetic platform that can be employed for CTX/PTT/PDT to treat cervical cancer. Here, the red blood cell (RBC) membrane decorated with targeting folic acid (FA) moiety was encapsulated with PEG-ylated WS_2_ NSs, which were in turn loaded with DOX (encapsulation efficiencies of 96.2%) and photosensitizer indocyanine green (ICG) (Figure 9). The synthesized scaffold exhibited increased circulation time, biocompatibility, and site-specific cellular uptake (>50% compared to control) for effective treatment. After 72 h at pH 5.2 and 7.4, the pH-based DOX release was 56.4% and 30.9%, respectively. With NIR irradiation for 30 min at pH 5.4, the DOX release was 30%, three times higher than that of the control. Based on the above promising results, the authors carried out in vivo studies and determined that RBC-membrane-coated NSs showed a 3.6-fold-enhanced half-time in the blood samples. Later, the anti-tumor activity was evaluated using various control and designed NSs, where, in the case of CTX monotherapy, the DOX release tumor inhibition rate (TIR) was 72.4%. In contrast, the combination therapy showed maximum liberation with a TIR of 95.4%, with almost complete ablation of cancer. Thus, this designed carrier can be efficiently employed as a multifunctional cargo for cancer theranostics [63].

**Figure 10:**
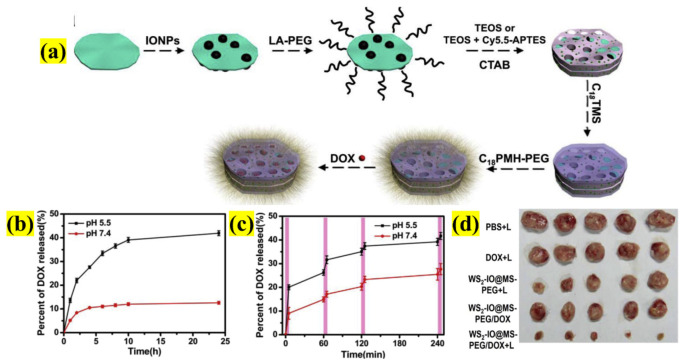
Schematic illustration of (**a**) the synthesis methodology of functionalized WS_2_ with iron oxide NPs and DOX encapsulated with PEG as a nanocarrier, (**b**) DOX-release-designed NSs in buffers at different pH values, (**c**) NIR-triggered release of DOX at different pH values (5.5 and 7.4), and (**d**) images of tumors collected after 14 days’ treatment of various mouse groups (adapted with permission from Reference [64], Biomaterials, published by Elsevier, 2015).

As 2D NSs provide an enormous opportunity for surface engineering, scientists have extensively tested their potential to treat cancer. Yang et al. designed a pioneering complex nanosystem comprising various nanostructures to exploit NSs multifunctionalities for enhanced surface functionalization for drugs, multimodal imaging, and mono/combined therapy (Figure 10). The NSs were strategically designed as follows: WS_2_ NSs were decorated with superparamagnetic iron oxide nanoparticles (SPIONs) and functionalized with mesoporous silica (Si), along with DOX. The whole system was finally encapsulated in PEG. Here, the inherent property of every material was studied carefully, for instance, WS_2_ NSs for PTT and computed tomography (CT) imaging, SPIONs magnetism for magnetic resonance imaging (MRI) as a T_2_ contrast, Si for effective DOX loading (0.5 mg/mL), and PEG for enhanced drug release, increasing blood circulation time, stability, and water solubility. The in vitro drug release at pH 5.5 and 7.4 was ~ 40% and 10%, respectively, at 24 h. In the presence of NIR irradiation, the DOX liberation from the nanosystem increased, particularly in acidic environments such as in cancer cell endosomes and lysosomes. The combined CTX/PTT treatment of 4T1 cells provided an improved therapeutic effect compared to monotherapies.

Furthermore, the half-life of the nanosystem in the tumor was evaluated using DOX fluorescence, which was more than >4.5 h, which is higher than that of many other DDSs. Finally, in the in vivo studies, the nanosystem in combined therapy, as expected, showed an enhanced therapeutic effect in cancer tissue, effectively reducing the tumor size. Thus, the findings demonstrated that the designed multifunctional nanosystem may greatly enable various chemical and biological modifications, demonstrating its advantages over WS_2_ NSs in cancer research [64].

### 2.4. Layered Double Hydroxides (LDHs)

Finally, to conclude our review on 2D NSs, we chose LDHs, which were discovered in the mid-20th century and have been employed for a wide range of purposes [65]. Most importantly, LDHs have been employed as antacids for human use. LDHs belong to a family of anionic ceramic materials with a positive charge. Therefore, two decades ago, they drew the attention of researchers, which led to their successful synthesis on the nanoscale. LDHs were effectively explored as one of the 2D alternatives for various biomedical applications [66]. These LDHs have extensive advantages such as facile synthesis methodology and intercalation chemistry, high stability, large surface area, reduced toxicity, enhanced cellular internalization and endosomal/lysosomal escape, pH-based sensitivity, biodegradability (acidic environment), anion exchange capacity, and easy tunability with metal particles [67].

These outstanding physicochemical properties qualify them for nano-bio convergence as a platform for therapy, imaging, and diagnostics [68]. Specifically, the field of cancer nanomedicine is searching for different and novel materials with functionalization behavior capabilities for use as a multifunctional nanostructure. Thus, the properties of LDHs are manipulated as with other 2D NSs, where they are decorated with various types of other nanostructures such as metals, polymers, biomolecules, etc. This led to the three main categories of LDHs in cancer theranostics: pristine, surface-modified, and composite-based. Usually, LDHs have been employed for drug delivery and gene/chemo/photo/immune/combined therapies to explore the principle, mechanisms, advantages, and limitations through various in depth studies [69].

**Figure 11:**
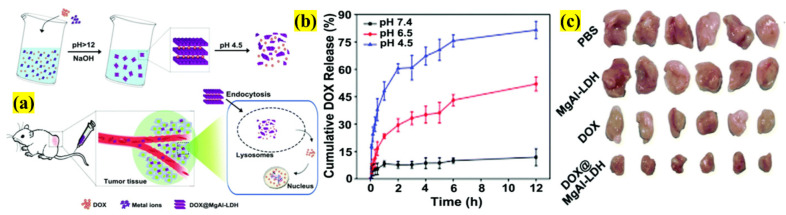
Schematic illustration of (**a**) the synthesis process of DOX-functionalized LDHs and employing them as nanocargo; (**b**) pH-based DOX release at 37 °C; (**c**) size comparison photographs of tumors obtained after treatment (adapted with permission from Reference [71] Journal of Materials Chemistry B, published by The Royal Society of Chemistry, 2018).

In this section, we explore LDHs as DDSs for mono or combined therapies with innovative functionalization, targeting ligands, and dopants, and the various, versatile methods of LDHs manipulation [70]. As mentioned earlier, one of the exemplary benefits of LDHs is the intercalation option, providing an enormous platform for bio-functionalization. Thus, LDHs are widely manipulated to provide various features. Recently, Hakeem et al. designed an LDHs-based DDSs platform by functionalizing with DOX to achieve targeting, internalization, and biocompatibility using the H22 hepatocarcinoma cell line (Figure 11). The synthesized carrier has a size of 220 nm and a surface charge of +18 mV with an LC and EE of 15% and 97.8%, respectively. The pH-based drug release at 7.4, 6.5, and 4.5 showed 7%, 46%, and more than 80% liberation, respectively, confirming that the release is favorable in acidic environments, so is suitable for tumor tissues. Additionally, the entry of the complex designed by various endocytosis pathways was seven-fold higher compared to the free drug. Furthermore, the functionalized LDHs significantly inhibited tumor growth by 64% compared to free DOX (36%) without affecting the animal body weight, thus providing a new strategy to obtain enhanced DOX efficacy in cancer therapy [71].

**Figure 12:**
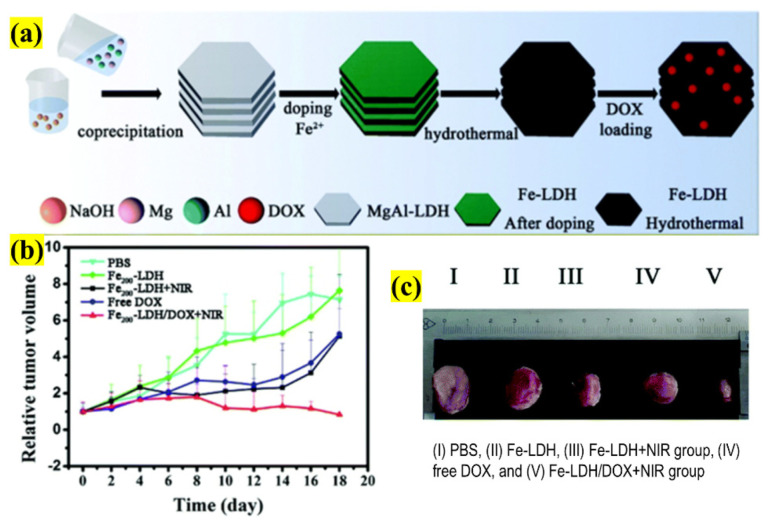
Schematic illustration representing (**a**) the design and preparation of Fe-doped LDH and functionalized with DOX as a nanotheranostic platform for combined therapy, (**b**) the tumor volume, (**c**) photographs of tumor tissue removed from various groups of mice after treatment in the presence or absence of NIR irradiation (adapted with permission from Reference [74], Biomaterials Science, published by The Royal Society of Chemistry, 2021).

Generally, researchers have considered doping with other elements to develop a nano/microstructure for its ultimate functionality or as an “all-in-one” platform [72,73]. This concept is highly recommended and has become a prevalent practice in cancer theranostics. For example, Guo et al. explored the doping of ferrous (Fe) ions with LDHs along with DOX loading to use it as a smart nanotheranostic for breast cancer (Figure 12). The main aim of this investigation was to employ the above-developed structure for combined CTX/PTT treatment using MRI guidance. The synthesized doped LDHs were 132.5 nm with 19.9% DOX loading, which was intercalated and decorated on the LDH plates. In order to determine the synergism of CTX/PTT, the nanostructure was incubated with cells and irradiated with an 808 nm NIR laser. The results showed that the release of DOX was exponentially enhanced and resulted in the complete ablation of cancer cells. The function of Fe ions was tested in MRI, confirming the concentration-dependent darkening effect of T_2_-weighted MR images (r^2^ value = 134.31 mM^−1^ s^−1^). Therefore, based on this result, the nanostructure is ideal for efficiently guiding both the diagnosis and prognosis of the tumor region, which could aid in treatment. Finally, based on the promising results, in vivo studies were conducted by injecting mice with the LDHs complex and irradiating them with a 808 nm laser (8 min, 1.5 Wcm^−2^ power density). The results showed that the synergistic effects were advantageous, with a maximum inhibition of tumor size achieved by only one exposure with no evidence of weight loss, justifying the doping concept by a facile fabrication methodology for advanced diagnostics and combined therapy [74].

The continuous R&D has tremendously advanced the field of DDSs in the past three decades with the employment of a wide range of nano-based organic, inorganic, and biomimetic materials [75,76]. This has led to various intrinsic, extrinsic, and dual-stimuli-responsive nanosystem designs being constructed to overcome drawbacks such as the EPR effect, quick drug burst, poor loading efficiency, sustained release, and finally, the renal clearance process [77]. Not all the formulations have been successful for clinical studies or use because the conventional nano-DDSs are usually made of various functionalizations and encapsulations of ligands, drugs, and polymeric membranes [78,79]. So, researchers have implemented a novel DDS concept by exploring a drug-eluting electrospun nanofiber as a carrier. These nanofibers provide an excellent surface area, facile bio-functionalization, substantial loading, and encapsulation efficiency. Additionally, it can be used for direct implantation at the tumor region [80,81].

**Figure 13:**
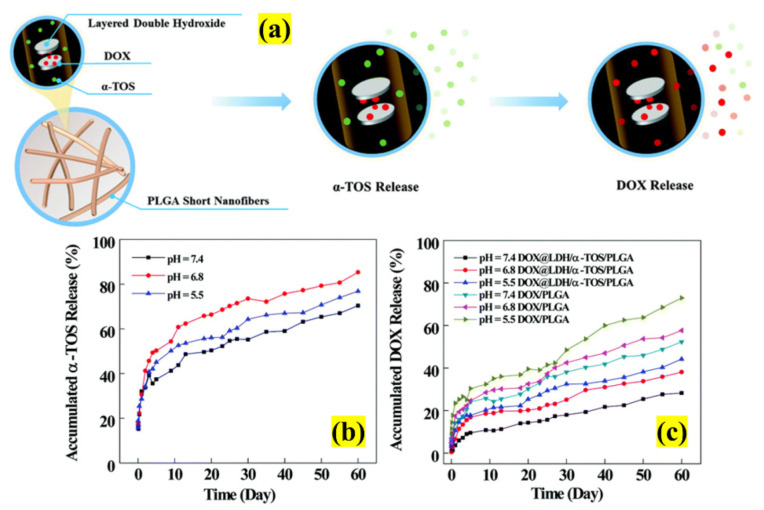
Schematic illustration of (**a**) the preparation of dual drug-loaded LDHs with PLGA nanofiber mats to treat MDR cancer cells; in-vitro release of (**b**) α-TOS and (**c**) DOX from the nanofibers (adapted with permission from Reference [82], New Journal of Chemistry, published by The Royal Society of Chemistry, 2021).

The 2D NSs have often been explored to take advantage of the properties drug-eluting nanofibers. Recently, LDHs with nanofibers were amalgamated and investigated for MDR cancer cells by Ma et al., as both share some similar characteristics [82]. This novel study aimed to develop an injectable multistage sustained dual DDS to overcome the problem of MDR and burst drug release by codelivering DOX and α-tocopherol succinate (α-TOS), as represented in Figure 13. Firstly, they fabricated LDHs-doped PLGA and drugs to produce a short electrospun fibers mat with a 17–24 μm length and a diameter of 830 nm. The in vitro study showed a pH-dependent release (28.26% at pH 7.4, 38.14% at pH 6.8, and 44.21% at pH 5.5) for a period of 60 days. More interestingly, the data showed the quick and slow release behavior of α-TOS and DOX, respectively. This strategy favors the extended diffusion of drugs to the target site and can highly effectively treat MDR cancer cells. This is made possible by the incorporation of drugs in LDs nanodisks with a fiber mat matrix. Therefore, the study confirmed the possibility of employing LDHs nanofiber mats as DDSs to treat MDR for an extended duration in long-term chemotherapy.

Cancer treatment depends on two main factors, space and time, which decide the efficacy of any formulation [83]. In order to provide space and time for treatment, numerous combinations and possibilities have been evaluated by researchers all around the world. Currently, nano- and micromotors have been designed with diverse driving forces such as fuel-dependent, free motors [84]. The motors’ missions encountered in the human body during treatment are survive, locate, operate, and terminate (SLOT) [85].

**Figure 14:**
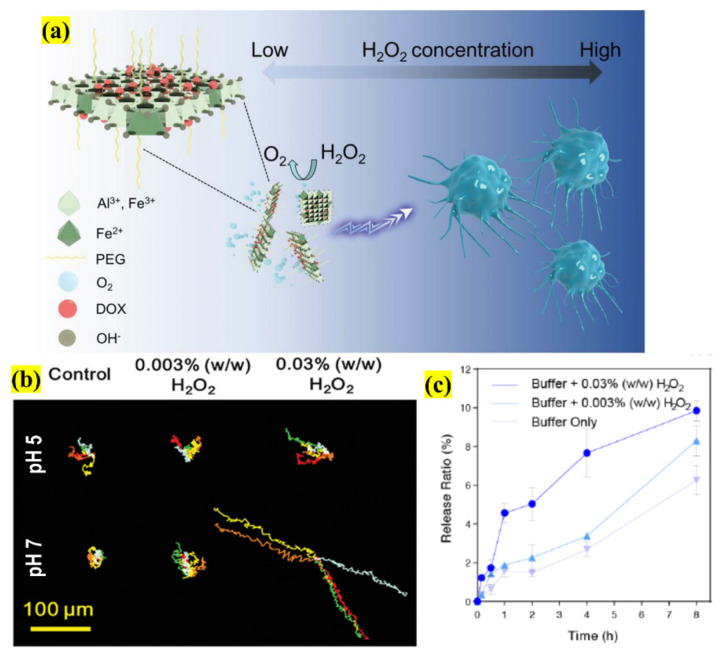
Schematic illustration of (**a**) the design of an LDHs 2D NSs stimuli-responsive nanomotor and its application in smart therapeutics using directional movement; (**b**) the trajectory movement of the nanomotor at different pHs and H_2_O_2_ concentrations; (**c**) DOX release profile from the nanomotor at pH 7 based on H_2_O_2_ fuel concentration for a period of 8 h (adapted with permission from Reference [14], Small, published by Wiley, 2020).

Centered on this innovation, Zhang et al. conducted an investigation by using an LDHs-based 2D NSs catalytic nanomotor as an efficient DDS in the TME using MCF-7 breast cancer cells (Figure 14). This nanomotor has an inherent characteristic of sustained release, large loading capability and surface area, and long-distance migration ability, which is highly suitable for therapeutics. The nanomotor was loaded with DOX via PEG-ylation (≈200 nm), and has an ability to migrate toward high concentrations of hydrogen peroxide (H_2_O_2_) fuel, demonstrating chemotactic properties. The produced nanomotor was studied for motion behavior using external stimuli; the results showed that both the migration speed and distance were considerably longer in the presence of H_2_O_2_ and an acidic environment, thereby confirming the dual way of nanomotor motion behavior by generating oxygen (O_2_) bubbles and serving as a propellant to stimulate. Surprisingly, the motor’s chemotactic motion was H_2_O_2_-concentration-dependent (0.003–0.03%) for effective propulsion. Finally, the DOX release was determined by an in vitro study with the cells at pH 7 and in the presence of the fuel. The results showed that the liberation was 7.67% ± 1.23% and 3.37% ± 0.14% for 0.03% and 0.003% H_2_O_2_, respectively, thus confirming the efficient cancer cell inhibition of the novel nanomotor design in the simulated TME even at a minimal dosage. So, this study reported 2D-NSs-based nanomotor performance, which opens possibilities for smart DDSs in the field of cancer nanotheranostics [14].

## 3. 2D Formulations with Biological Barriers (BBs)

Nano-based DDSs show exemplary behavior in cancer treatment by delivering the drugs from the administration site to the target region, thereby overcoming the major drawback of conventional drug carriers. However, they must overcome the major obstacle posed by complex BBs to achieve efficient treatment of disease. Therefore, currently, nanocarriers are being strategically designed to overcome such obstacles [86]. This review discusses the early-stage advances of various 2D NSs drug cargoes; thus, we briefly explain how these next-generation formulations are efficiently designed based on quantitative–structural activity relationships (QSARs) with the aim of overcoming various BBs, effectively delivering selective uptake, and releasing drugs in the tumor microenvironment (TME) [87].

Based on the three following requirements, these objectives can be achieved by evaluating NSs using cell-culture, animal, or 3D models as the standard reference of designed NSs to conduct mechanistic research, perform biodistribution studies with a safe-by-design approach, and finally construct a multifunctional platform to manipulate BBs. These approaches will help with predicting both the therapeutic potential and toxicity of the formulation [88]. The vast surface area of 2D NSs is highly interactive with the immune system, which plays a vital role in BBs, affecting the delivery of drugs to the targeted site. So, in recent years, researchers have shown keen interest in studying the nano-bio interface of nanomaterials. Interface studies depend on protein corona formation and various physicochemical properties, including biocompatibility, surface functionalization, dissolution in the body fluids, and the Trojan horse mechanism [89].

Most of the 2D NSs designs overcome or avoid the effect of BBs using some important modification principles [90] such as:

(i) Opsonization: To overcome this process, most of the NSs-based investigations have focused on polymeric or biomimetic cell membrane coating technology to encapsulate the functionalized NSs.
(ii) Hemorheological limitations: The size, surface charge, and geometry play a vital role in blood fluid dynamics and circulation. So, researchers have designed NSs-based formulations with size ranging from 100–250 nm (maximum 300 nm) with multifunctionality. Considering surface charge, most NSs have a negative charge (around −20 mV), extending the circulation time. Lastly, a non-spherical structure that relies on margination and adhesion maximizes after administration seems favorable compared to spherical-shaped nanostructures.
(iii) Cellular internalization is enhanced by decorating cationic moieties on the NSs surface with polypeptides such poly-l-lysine (PLL), PEI, and PAMAM for internalizing, and for endosomal or lysosomes compartmentalization.

Therefore, the advances in 2D NSs as drug delivery agents is moving along a determined path showing more promise than any other nano-based formulations. Thus, the investigations discussed in this review show that 2D therapeutic formulations are either aid in decreasing tumor volume or inhibit tumor growth with no reoccurrence. Regarding other BBs, in the current scenario, 2D NSs research is in its infancy, requiring more in-depth study, which will be carried out in the future.

## 4. Conclusions and Perspectives

In summary, this review provided a detailed summary of the current advances in 2D NSs in therapeutics for different types of cancer. We also discussed the various polymeric encapsulation, drug decoration, and synergistic effects of CTX/PTT for efficient drug release. The 2D NSs carrying suitable nanocargo show vast potential to meet and overcome the current cancer treatment challenges. Primarily, the attractive properties of NSs provide the opportunity to focus on various challenges, such as drug resistance, recurrence, and metastasis. Therefore, the field of NSs-based cancer nanomedicine is emerging and evolving more rapidly than any other nanostructures developed lately.

In most cases, NSs have been mainly considered as a multifunctional system with a wide range of surface-functionalized moieties because of NSs’ large surface area and facile tailoring features. NSs also qualify as a nanosystem because of their biodegradation and biocompatibility aspects. Based on these behaviors, NSs are broadly considered a versatile platform for CTX/PTT/PDT therapies and as an imaging and diagnostic agent. Most importantly, various investigations discussed in this review concentrated on size, which is usually a critical factor that must be maintained in a precise range considering the EPR, making them suitable for various applications. Thus, the novel design of various NSs on a laboratory scale resulted in less cytotoxicity and no recurrence of cancer, which were confirmed by in vivo studies. These advantages could pave the way for clinical translation. Additionally, to address the shortcomings of conventional therapy, different forms of scaffolds or matrices have been employed for stimuli- and response-based, site-specific release, decreasing the concentration to minimal in vital organs.

The above investigations show that these NSs may be an excellent next-generation nanomedicine for therapeutics, which may save patients’ lives, reduce the adverse side effects of treatment, and improve the quality or their health. Finally, the choice of 2D nanomaterials for biomedical applications is mainly based on their toxicity and potential application. Even though the above-explained NSs seem biodegradable and biocompatible, these features depend on a considerable number of factors that should be considered carefully before employing them in the biomedical sector, for example, synthesis technique, shape, surface functionalization compounds, size, dispersion and oxidation state, dosage, and route of administration. Most importantly, the significant advantage of NSs’ surface area could be disadvantageous because of increased interactions with blood and its components, which may lead to precipitate formation (serum proteins or RBC) in the case of in vivo administration. This could result in the activation of the immune system, so main quest lies in selecting NSs for cancer therapeutics or any other biological applications.

Nonetheless, NSs, in general, have inherent unique physiochemical properties, providing their advantages by maintaining a common platform for 2D materials. So, the selection of material for therapeutics must be based on two critical factors:

(1) Synthesis method: For all the above-discussed NSs, a high-yield, cost-effective, large-scale fabrication method is necessary to produce a tightly controlled size, thickness, and stability.
(2) Toxicity: All four different NSs are biocompatible with either low or negligible cytotoxicity even at higher concentrations, and are biodegradable in biological environments.

Therefore, when comparing all the NSs discussed in this review, we speculate on their appropriate selection by providing a brief description to give the readers an idea of the basis upon which 2D NSs can be chosen. As this field of research is still in the early stages, providing stronger evidence of specific characteristics is not possible. Thus, the explained criteria are based on in vitro and in vivo studies. The toxicity testing has been conducted by researchers in terms of size, shape, concentration, and cell type. For instance, in the case of BP NSs, the cytotoxicity is directly proportional to size. So, factor linked with the synthesis technique plays a vital role in determining the application of NSs in biomedicine.

Hence, to provide an idea, we can consider G, one of the widely investigated 2D NSs in this decade. G has a significant impact on toxicity by affecting various signaling pathways, destroying the mitochondrial membrane and causing dysfunction, leading to ROS generation and accumulation in lungs, which lead to pulmonary lesions, edema, and inflammation. Thus, regarding the in vitro studies, 2D NSs have been successful, but when moving in in vivo studies, the scientist’s choice of material could be narrowed to overcome the disadvantages mentioned above. This is one reason why most nanomaterials are celebrated in research but do not proceed to the following stages toward clinical translation. So, to extend the possibilities of their use, in-depth mechanistic and computational approaches are necessary to determine the NSs–bio interface and its impacts. Finally, we hope this review provides comprehensive outline of the various 2D NSs and their potential as nanocarriers in the oncological sector.

## Figures and Tables

**Table 1:** Some of the recent advances in various NSs as DDSs along with surface functionalization, targeting ligand, drugs employed, their characteristics, treated cancer types, days, and their interpretations for multimodal cancer therapy.

2D NSs	Surface Functionalization	Targeting Ligand		Drug	LC (mg or μg)/EE (%)	Size (nm)	ζ Potential (mV)	Synergistic Effect	Cancer Type	Treatment Days	TGI Rates (%)/Observation after the Treatment	Reference
BP				DOX	950% (in wt)	281 ± 9.5	1.5	CTX/PTT/PDT	Breast cancer (4T1 cells)	14	95.5%	[91]
	PAMAM	HA			95%	291 ± 4.17	−5.83 ± 2.31	CTX/PTT			Reduction in tumor size	[92]
		CaP			53.6%	181.5	−15.5	CTX	Breast cancer (MCF-7 cells)	18	85%	[93]
	HA			MTX	2.6%	276	−18	CTX/PTT	Breast cancer (4T1 cells)	10	Inhibit tumor growth	[94]
MoS_2_	PEG			DOX	~69%	150	−7.17 ± 1.8		Sarcoma (S180 cells)	14	Decrease in tumor volume	[95]
		CuS			162.3 mg	114.5	−5.3 ± 1.5		-			[96]
	PEI, PEG, α-LA	FA, BSA			185 mg	196	6.8		Breast cancer (MCF-7 cells)	20	Inhibit tumor growth	[97]
	Liposome				104.4%	250	−38.26		Breast cancer (4T1 cells)	2		[98]
	CS, CMC, SPIONs				95.69%	429.07 ± 3.538	−59.01 ± 3.629			14		[99]
Ti_3_C_2_		HA			84.2%	178 ± 32.4	−20.71 ± 1.5	CTX/PTT/PDT	Colorectal cancer (HCT-116 cells)	16	Tumor disappearance and no metastasis/recurrence	[100]
	CP			MET	96.2%	310	−20		Breast cancer (MDA-MB-231 cells)			[101]
	PDA, PEG-GNRs			DOX	95.88%	250	−22.1 ± 1.2	CTX/PTT	-			[102]
LDHs	AuNPs		FA-TCS		94.6%	100–180	−52.4		Breast cancer (MCF7 cells)	-	Increased % of cell death at the G0/G1 phase	[103]
	BSA			5-FU	56.4% ± 7.4%	172.4 ± 10.6	−14.0	CTX	Colorectal cancer (HCT-116 cells)	18	Significant and effective inhibition of tumor growth	[104]
				ABX	93.2% ± 5.2%							
	ICG			-	99.6% ± 0.1%	38.8 ± 1.8	33.9 ± 0.9	CTX/PTT/PDT	Breast cancer (4T1 cells)	12		[105]
				5-FU and CD-siRNA	22.60% (in wt)	89	38.9	CTX	Breast cancer (MCF7 cells)	-	Suppresses cell growth	[106]
	Gal			Cur	31%	116.1 ± 35.9	10.0 ± 0.7		Hepatocellular cancer (HepG2 cells)	-		[107]
	BSA			5-FU	-	41.2 ± 5.4	-	CTX/PTT	Colon cancer (HCT-116 cells)	24	Induces cancer cell death with no sign of recurrence	[108]
				MTX	42% ± 0.25%	104	-	CTX	Cervical cancer (C33A cells)	4	Tumor growth suppression	[109]
	FA			DOX	3.6 mg/mg (*w*/*w*)	60–100	−3.2		Oral epidermal cancer (KB cells)	-	Predominant apoptosis of cancer cells	[110]
	PDA				-	125	−14.61	CDT/PTT	Lung cancer (A549 cells)	-		[111]
	F-CDs				212.6 μg/g	137	39.7	CTX	HeLa cells	-	Enhanced cytotoxicity	[112]

## Data Availability

Not applicable.

## Abbreviations

α-TOS	α-tocopherol succinate
2D	Two-Dimensional
3D	Three-dimensional
5-FU	5-fluorouracil
ABX	Abraxane
ADR	Adverse drug reaction
ATP	Adenosine triphosphate
AuNPs	Gold nanoparticles
BSA	Bovine serum albumin
CAT	Catalase
CaP	Calcium phosphate
CD-siRNA	Cell death-siRNA
CDT	Chemodynamic therapy
CIS	Cisplatin
CS	Chitosan
CT	Computed tomography
CTX	Chemotherapy
Cur	Curcumin
CuS	Copper sulfide
CMC	Carboxymethyl-cellulose
DACH	Oxaliplatin
DDSs	Drug delivery systems
DNA	Deoxyribonucleic Acid
DOX	Doxorubicin
EE	Encapsulation efficiency
EPR	Enhanced permeability and retention
F-CDs	Folate-carbon dots
FA	Folic acid
FDA	Food and Drug Administration
Fe	Ferrous
G	Graphene
Gal	Galactose
GNRs	Gold nanorods
H_2_O_2_	Hydrogen peroxide
HA	Hyaluronic acid
ICG	Indocyanine green
IM	Imatinib mesylate
LA	α-lipoic acid
LC	Loading capacity
LDHs	Layered double hydroxides
M	Million
MDR	Multiple drug resistance
Mel	Melanin
MET	Metformin
MRI	Magnetic resonance imaging
MTX	Methotrexate
NIR	Near-infrared
NSs	Nanosheets
O_2_	Oxygen
PAMAM	Poly(amidoamine)
PC	Phosphatidylcholine
PC	Prostate cancer
PDA	Polydopamine
PDT	Photodynamic therapy
PEG	Polyethylene glycol
PEI	Polyethylenimine
PEITC	Phenethyl isothiocyanate
Pgp	P-glycoprotein
PDA	Polydopamine
PLGA	Poly(lactic-co-glycolic acid)
PLL	Poly-L-lysine
PTT	Photothermal therapy
QSAR	Quantitative-structural activity relationship
R&D	Research and development
RBC	Red blood cells
ROS	Reactive oxygen species
RV	Resveratrol
SLOT	Survive locate operate and terminate
SPIONs	Superparamagnetic iron oxide nanoparticles
TCS	Thiolated chitosan
TGI	Tumor growth inhibition
TIR	Tumor inhibition rate
TMDCs	Transition metal dichalcogenides
TME	Tumor Microenvironment
US	United States
UV	Ultraviolet
wt	Weight
